# Stressors and support system among parents of neonates hospitalised with systemic infections: qualitative study in South India

**DOI:** 10.1136/archdischild-2020-319226

**Published:** 2020-11-11

**Authors:** Shruti Murthy, Vasudeva Guddattu, Leslie Lewis, Narayanapillai Sreekumaran Nair, Hinke Haisma, Ajay Bailey

**Affiliations:** 1 Department of Data Science, Prasanna School of Public Health, Manipal Academy of Higher Education, Manipal, India; 2 Department of Paediatrics, Kasturba Medical College, Manipal, Karnataka, India; 3 Department of Medical Biometrics and Informatics (Biostatistics), Jawaharlal Institute of Postgraduate Medical Education and Research, Puducherry, Tamil Nadu, India; 4 Population Research Centre, Faculty of Spatial Sciences, University of Groningen, Groningen, The Netherlands; 5 Department of Human Geography and Spatial Planning, Faculty of Geosciences, Utrecht University, Utrecht, The Netherlands; 6 Transdisciplinary Centre for Qualitative Methods, Prasanna School of Public Health, Manipal Academy of Higher Education, Manipal, Karnataka, India

**Keywords:** infectious diseases, neonatology, qualitative research, patient perspective, multidisciplinary team care

## Abstract

**Objective:**

To explore stressors and support system for families with a neonate admitted with a systemic infection.

**Design:**

Qualitative study using in-depth interviews (IDIs), based on principles of grounded theory.

**Setting:**

A busy level III neonatal unit of a tertiary care teaching hospital in coastal Karnataka, India, between May 2018 and January 2019.

**Participants:**

Parents and accompanying attendants of neonates admitted to the neonatal unit with one or more systemic infections.

**Methods:**

Using purposive sampling, semi-structured IDIs were audio recorded, transcribed verbatim and a thematic analysis was performed.

**Results:**

Thirty-eight participants were interviewed, lasting between 30 and 59 min. Babies’ hospitalisation with sepsis was an unprecedented, sudden and overwhelming event. Stressors related to uncertainties due to the information gap inherent to the nature of illness, cultural rituals, financial constraints, barriers to bonding and others. Parents reported experiencing insomnia, gastric disturbances and fatigue. Support (emotional and/or financial) was sought from families and friends, peers, staff and religion. Availability and preference of emotional support system differed for mothers and fathers. In our context, families, peers and religion were of particular importance for reinforcing the available support system. Participant responses were shaped by clinical, cultural, financial, religious and health service contexts.

**Conclusion:**

Designing a family-centred care in our context needs consideration of stressors that extend beyond the immediate neonatal intensive care unit environment and interactions. Understanding the influence of the nature of illness, financial, familial and cultural contexts helps identify the families who are particularly vulnerable to stress.

## Abstract (precis)

In the S Indian context, support for parents whose child is admitted to a neonatal unit with sepsis needs to go beyond the NICU and include, extended family, cultural and financial issues as well as NICU practices.

## Introduction

Neonatal intensive care unit (NICU) hospitalisation is traumatic for neonates and their entire family. Families of hospitalised preterm/low-birthweight neonates experience stress, insecurity and alienation.^1 2^ However, sepsis in the baby—a time-critical, devastating bloodstream infection with an unpredictable clinical course—poses different challenges for a family compared with babies who are not seriously ill.^3–6^ It can range from a localised infection to life-threatening manifestations.^3 4^ Complications (eg, septic shock and cardiopulmonary arrest) can progress suddenly and rapidly leading to multiorgan dysfunction and death, despite corrective measures.^3^ This situation can be frightening for parents, with stressors affecting different fronts of their lives.^4–6^ Families of infected/seriously ill babies are subject to health inequalities, face acute stressors, worry constantly about devastating consequences and continually have feelings of inadequacy.^5 7 8^ Moreover, neonates of stressed parents are at the greatest risk of cognitive and behavioural problems compared with healthy neonates.^9–11^

Research on NICU experiences, particularly from high-income countries (HICs), has driven a paradigm shift in neonatal care design, giving rise to concepts such as ‘family integrated care’, ‘family-centred care’ (FCC) and ‘neonatal intensive parenting unit’.^12–15^ These studies have informed innovative policy guidelines for incorporating mental health professionals, peer support and communication changes in neonatal care.^16–18^ However, research in India exploring families’ psychosocial needs during NICU hospitalisation is largely quantitative^19–22^; qualitative research is scarce,^23^ with none focusing on sepsis. Numerically ‘scoring’ parents’ experiences does little to capture in-depth experiences of familial needs and contexts. Understanding families’ cultural contexts (eg, religious and daily life practices) and support systems is essential for understanding parental needs and effectively using their capabilities in designing and supporting FCC.^24–26^

Despite policy recommendations on introducing FCC in Indian public healthcare, progress has been slow.^22 27^ Neonatal care continues to be technology-driven and provider-centred, with limited parent involvement.^22^ Considering that India bears the highest global burden of neonatal sepsis,^28^ this exploratory study was conducted in coastal South India to understand the (1) stressors and (2) sources of support for parents and accompanying attendants having a baby admitted for the treatment of sepsis in a private tertiary care level IIIC NICU.

## Methods

The study protocol (online supplemental file 1) supplements this section with additional details. A qualitative study using in-depth interviews (IDIs), guided by the principles of grounded theory,^29 30^ was conducted between May 2018 and January 2019 in a level IIIC NICU of a tertiary care teaching hospital in a coastal South Indian district. We included parents and grandparents (1) having babies (0–28 days) admitted to the study site for treatment of a systemic infection and (2) providing written informed consent. A purposive sampling was used to recruit participants immediately or within the first few days after diagnosis to capture and understand their experiences as soon as they knew of their baby’s condition. Exclusions included (1) parent/spouse unavailable at the study site during the data collection period and (2) parents with babies who were in the midst of treatment.

10.1136/archdischild-2020-319226.supp1 Supplementary data

Participants were asked to describe their NICU experiences in terms of stressors and support system. A pilot study conducted on five participants refined the semistructured IDI guide (online supplemental file 2) to fit our research inquiry. Audio-recorded interviews were conducted in a participant-preferred language, location and time.

10.1136/archdischild-2020-319226.supp2 Supplementary data

A thematic analysis was performed (Atlas.ti). Interviews were transcribed and anonymised, and field notes were typed following each interview. Coding and subsequent categorisation were done in two cycles,^29^ and data were organised to list the participant-reported stressors and support systems. We inductively derived our categories conceptually^29 30^ under the two themes of ‘stressors’ (table 1) and ‘support system’ (table 2) to address our two research objectives. Exploration of concepts in subsequent interviews guided data saturation and identification of disconfirming sources.^30–32^

**Table 1:** Coding scheme for a category under the theme ‘stressors’

Category	Subcategory	Code	M	F	GP
Uncertainties due to information gap Participants did not know or understand aspects related to sepsis or its hospitalisation, which led to uncertainties.	Unfamiliar disease	What has happened to baby	x	x	x
		Reason for infection unknown	x	x	x
		Why my baby	x		x
		Why sudden admission	x	x	x
		Chances of survival unknown	x	x	x
		Discharge date unknown	x	x	x
		Why breast milk not given		x	
		If treatment is working		x	
		False assurance	x	x	x
		Scary terminologies	x	x	
		Will baby be disabled	x	x	x
		Why so many doctors around the baby	x		x
	Sudden deterioration in baby’s condition	Sudden vomiting	x	x	x
		Vomiting milk since morning	x	x	
		Weight loss	x	x	x
		Stopped drinking milk today	x	x	x
		Sudden stomach swelling	x	x	x
		Stomach swelling worse today	x		
		Suddenly requires breathing support	x	x	x
		Requires ventilator today	x	x	
		Not active today	x	x	x
		Suddenly drowsy		x	x
		Suddenly seems limp		x	x
		Sudden shaking of hands	x	x	
	Cost-related	Cost estimate unknown		x	x
		No transparency in cost increase		x	x
		Reason for stalled financial scheme unknown		x	x
		‘Who to ask for money?’		x	x
		Unsure on financial scheme—availability	x	x	x
		Unsure on financial scheme—use	x	x	x
Financial constraints ‘Relates to participant’s struggles in arranging money to pay for medical and non-medical costs related to baby’s hospitalisation’	Inaccessible financial support	Baby not named		x	x
		Insurance applies to either mother or baby	x	x	x
		Inadequate information		x	x
		Contrasting information		x	x
		Stalled schemes		x	x
	Lack of adequate resources	Limited or no savings		x	x
		No income		x	
Barriers to bonding Pertains to barriers that hindered participant–baby bonding		Inaccessible visiting hours		x	x
		Postpartum movement restrictions	x	x	x
		Mother unwell	x	x	x
		Mother in a different hospital		x	x
Unfamiliar NICU environment Pertains to first-time experience with sights and sounds in NICU and the hospitalised baby		Tubes and pricks—baby	x		x
		Oxygen mask	x		x
		Beeping sounds	x		x
		Machine/incubator	x	x	x
		Inadequate breast milk	x		x
		Sick baby	x	x	x
		Baby in ventilator/machine	x	x	x
		Pumping breast milk	x	x	x
		Baby’s distress	x	x	x
Spouse/parent health Pertains to participants’ stress regarding their spouse’s health (father/mother) or parent’s health (grandparent)	Physical	Insomnia		x	x
		Disturbed sleep	x	x	x
		Insufficient sleep	x	x	x
		Gastritis		x	
		Chest pain		x	
		Back pain	x		x
		Leg pain	x		
		Weakness	x		
		Dizziness	x		
		Fatigue	x	x	
		Not eating			x
	Emotional	Stressed	x	x	x
		Crying	x	x	x
		Not talking			x
Mute spectator in distress Pertains to participants’ inability to		Reduce baby’s pain	x		x
		Empathise with pain	x	x	x

F, father; GP, grandparent; M, mother; NICU, neonatal intensive care unit.

**Table 2:** Coding scheme for a category under the theme ‘support system’

Category	Subcategory	Code	M	F	GP
Family and friends	Emotional	‘Don’t worry, baby will be fine’	x	x	
		Expressing feelings	x		x
		Crying	x		x
	Financial	Savings from father, in-laws and paternal uncle		x	
		Lease on assets from father		x	
		Income and savings from brother		x	
		Interest-free loan from friends		x	
Peers	Sepsis-related experiences	Familial background information	x		
		Breastfeeding problems	x		
		Baby’s condition	x		
		Feelings of guilt and shame	x		
	General hospitalisation experiences	Postpartum recovery	x	x	x
		Fatigue	x		
		Husband’s/parent’s background	x		x
		Selective information sharing		x	
		Financial support		x	x
		Own background	x	x	x
		Hospital facilities	x	x	x
Staff	Doing their job	Trusting staff	x	x	x
		Observing staff do their duty		x	
		Caring despite being busy		x	
		Running around without rest		x	
	Trouble shooting	Breastfeeding challenges	x		
		Kangaroo mother care	x		x
		Pumping breast milk	x		x
	Knowledge and competency	Understand baby’s condition	x		
		Understanding machine	x		
		Understanding sights and sounds	x		
		Learning to operate machine	x		
		Postpartum health and nutrition	x	x	
		Baby care	x		
Religion		Undertaking ‘Harke’ (vows)		x	x
		Placing faith in God	x	x	x
		Donating to religious cause		x	x
		Praying to God	x	x	x

F, father; GP, grandparent; M, mother.

Stressors were coded by identifying factors that the participant described as making them feel strained, tensed or something which they did not prefer to encounter (table 3). Support was described in terms of what/who supported them emotionally and financially/reduced their stressful emotions (table 3). Physical health associated with the themes was also coded. We ensured trustworthiness by instituting measures for credibility, confirmability, dependability, transferability and positionality (table 4).^33 34^

**Table 3:** Emotions and feelings attributed to a stressor and support

Stressor	Support system
Aatanka (terror), bhaya (fear), hedarike (scared or frightened), sankata (problem), vikopa, kopa (rage or anger), dukkha (sadness), manasige navvu (hurting/painful to the mind), bejaru (feeling bad), sakkath chinte (worry), torture, anxiety, tension, stress, distress, helplessness, disappointment, hopelessness, frustration, hurtful, painful	Khushi (happiness or joy), cheerful, confidence, hopeful, chennagi ansodu (feeling good), feeling positive, don’t feel bad anymore, not worried, shanti (relieved, calm or at peace).

**Table 4:** List of measures taken to ensure rigour in our study

Criteria	Measures taken in our study
Credibility and confirmability	Triangulation of data from multiple sources of data collection. Audio-recording of interviews to retain information. Participant verification of handwritten interviews. Continuous and active immersion in data. Multidisciplinary team (experts in subject and methods) for Interpretation of data (SM, AB, HH and LL). External review of findings. Minimising subjective interpretations by adequate researcher (SM) training and experience in qualitative research methods and analysis. Checking coding cycles by a second qualitative expert (HH).
Dependability	Detailed report of research plan, implementation and challenges. Thick description of results written before preparation of manuscript. Provision of explicit reporting of analysis plan, participants quotations and emotions.
Transferability	Detailed protocol (online supplemental file 1) to report study design and context. Study limitations reported for further understanding of study settings.
Positionality	About the interviewer (SM) Is an unmarried female PhD student. Was in the third year at the time of interviewing. Is external to NICU and not a member of clinical NICU team. Is qualified in Bachelor of Dental Surgery and Master of Public Health. Has research experience since 2011. Has adequate training and experience in qualitative research methods and analysis.

NICU, neonatal intensive care unit.

## Results

Thirty eight of 47 participants took part in the study (19 mothers, 15 fathers, 4 grandparents; online supplemental file 3 and table 5). Three parents were interviewed as a couple. Three grandparents accompanied the mother and one grandfather accompanied a father. IDIs lasted between 30 and 59 min. Nine repeat interviews were conducted. The interview was handwritten for one father and mother as they denied permission for audio-recording. The experiences have been described under the two themes of ‘stressors’ and ‘support system’ to address our research objectives. Mothers and grandmothers frequently cried and could not vocalise their despair. Mothers hesitated to express themselves in front of their husbands. Participants reported their baby’s hospitalisation to be an emotionally and financially shocking and unprecedented event (box 1, quotes 1 and 2) for them and their families.

10.1136/archdischild-2020-319226.supp3 Supplementary data

Box 1 **Unprecedented situation and sudden deterioration in baby’s condition**

**Unprecedented situation**

Quote 1: So after the birth of the baby, we will be expecting a healthy baby. We will not be knowing something that is related to all this [infection]… [we] would not be knowing if it would not have happened to us or our parents. We have a belief that [baby care] will happen easily and it will be economical. But when such a thing happens, it becomes a headache, a big problem and heavy burden for people like us, because [we will] not be having any information. (Father, 37 years, neonatal sepsis)

Quote 2: We had not heard of anything like this. And never have we seen a small baby having such a condition to be kept in the machine. It was not like this in our times. We have played with many babies and all babies were healthy. We have not seen all of this at all. All our children grew well, we did not have a sight of all this. God should not give anyone this this trouble. (Grandmother, 44 years, neonatal meningitis)

**Sudden deterioration in baby’s condition**

Quote 3: At one time they say, “[The baby] is getting better, let’s see”. At another time, they say, “There is danger”. Why? What happened suddenly? I am is so frightened. (Mother, 33 years, neonatal meningitis)

Quote 4: They [doctors] are saying now that the intestines are not working…and it’s started vomiting now. Something has happened. They are not telling us, something has suddenly happened. (Grandfather, 57 years, sepsis with necrotising enterocolitis)

**Table 5:** Participant characteristics (N=38)

Characteristic	Category	Mother n=19	Father n=15	Grandparent n=4	Total N=38
Age range (in completed years)	21–30	14	7	–	21
	31–40	5	8	–	13
	41–50	–	–	2	2
	51–60	–	–	1	1
	61–70	–	–	1	1
Occupation type	Homemaker/unemployed	17	1	1	19
	Business	–	5	1	6
	Fixed-term employees	1	5	–	6
	Daily wage labour	1	2	1	4
	Agriculture/fishery	–	1	1	2
	Permanent employees	–	1	–	1
Type of family	Joint	11	4	–	16
	Nuclear	15	7	–	22
Residence in the same town of study site		4	3	–	7
Admission	Outborn	–	–	–	20
Type of pregnancy	Lower segment caesarean section	18	8	–	26
	First pregnancy	14	5	–	19
	Abortions—1	2	1	–	3
	Abortions—2	2	2	–	4
	Abortions—3	–	1	–	1
	Neonatal death	–	1	–	1
	Live child at home	–	–	–	5
Type of systemic infection	Neonatal meningitis	4	1	–	5
	Neonatal sepsis	19	8	–	27
	Neonatal pneumonia	2	3	–	5
	Septic arthritis	1	–	–	1
	More than one systemic infection	1	1	–	2
Sex of neonate	Male	15	10	4	29
Previous experience with sepsis (families and friends)	Requiring admission	–	–	–	0
	Not requiring admission	–	–	–	0
Previous intensive care experience		–	–	–	0

### Theme: sources of stress during baby’s hospitalisation due to sepsis

Stressors were described by participants as sources that caused anxiety, tension, strain and anguish (table 3) when dealing with their baby’s hospitalisation. Participants typically reported more than one emotion for each stressor. Stressors related to uncertainties (due to nature of illness and finance), cultural practices, financial constraints, barriers to bonding and others. Grandmothers typically mentioned that ‘no one should have to face a situation like this’ and they questioned why ‘God was showing them such a day’.

1. **Uncertainty due to information gaps**: parents were disturbed and anxious when faced with uncertainties regarding their baby’s well-being or NICU-related costs.
  - Baby’s survival and well-being.
    - Sudden bouts of deterioration in baby’s condition (box 1, quotes 3 and 4): participants were frightened when they did not understand suddenly why the baby deteriorated when the baby seemed to be recovering (eg, abdominal distention, vomiting, weight loss and drowsiness; table 1). This led to feelings of shock, confusion, misunderstanding, guilt and sadness among all participants.
    - Baby’s condition, treatment and outcome (box 2): mothers repeatedly blamed themselves for their baby’s condition (quote 1). Additionally, they expressed an unmet need of understanding why it happened to their baby, if other babies faced such illnesses (quote 2), what it meant for their baby to have an infection and why no guarantee was given for the baby’s survival or discharge date (quotes 3 and 4), why they were separated from their babies and why certain treatment decisions were made by the staff (quote 5). The anxiety and fear resulted in some fathers perceiving a loss of trust in the staff (quote 6). However, participants typically hesitated to ask for more information from the staff (quote 7).
  - Financial uncertainty (box 3, quotes 1–3): many outborn admissions were referred ‘urgently’ to the study site because (1) baby did not improve clinically despite treatment or (2) infrastructural problems (also confirmed from NICU staff and medical records). Participants reported receiving no information, from referral facilities regarding the type of setting they would encounter financially. Fathers and grandparents, particularly of outborn babies, felt unprepared and helpless when their need for ‘timely, transparent and accurate cost-related information’ was unmet. Unemployed mothers were unaware of the treatment costs and mentioned their husbands handling financial decisions.
2. **Financial constraints** (box 3, quotes 4–6): families who lacked health insurance and faced inaccessible financial support had to bear all costs. With limited money at hand, fathers and grandparents struggled to source money ‘by hook or crook’. A manual labourer recalled preventing his recently delivered sick wife from seeking medical help as the insurance covered only the baby’s costs. Fathers faced conflicting choices of having to remain on site at the NICU, go offsite to earn money or avail financial assistance.
3. **Cultural rituals** (box 3, quote 7): some mothers mentioned feeling stressed about cultural practices which they believed were responsible for their baby’s infection that ultimately required hospitalisation. Participants also voiced their worry if hospitalisation meant a delay in observing cultural rituals/ceremonies for their baby.
4. **Barriers to bonding** (box 4, quotes 1–4): some mothers and babies were hospitalised in separate hospitals postdelivery (eg, outborn early-onset sepsis). Such mothers experienced ‘uncontrollable rage’ as they felt insecure and desolate, being unable to look after their babies immediately after birth. Fathers were frustrated at inflexible workplace and NICU visiting policies, which prevented them from visiting the baby for days. Grandmothers reported mothers getting hysterical when they could not see their baby for a week. In contrast, a couple recalled how their antenatal provider referred them to our study site before delivery (as opposed to just the baby after delivery), in order to prevent separation of mother and baby (quote 4).
5. **Others**: this included having inadequate breast milk, being a spectator during baby’s distress, struggling to manage additional roles without support, individual or spouse’s disturbances in health (table 1).

Box 2 **Baby’s condition, treatment and outcome**

Quote 1: All I feel is tension. I have lost all my happiness. My baby…not sure what may happen. There is panic regarding what may happen to my baby. What is this condition I do not understand…What a horrible mother I must be to have given it to my baby in the womb. No one should go through this. Why is God showing us such a day? [weeping]. (Mother, 28 years, neonatal sepsis)

Quote 2:

GF: Madam, do you know if such babies are admitted here? Does this happen to other babies or is our baby the only one here like this? I want to understand if there are other babies like ours in this hospital…

F: Yes, we are very worried, we want to know if this has happened to other babies before.

(Father (F), 28 years, and grandfather (GF), 57 years, neonatal sepsis with necrotising enterocolitis)

Quote 3: I am waiting when I can go back. But I am not getting any information on when my baby will get better. They have been trying for quite a while. They say that there is problem in the…what do you call that? (points to the throat). They don’t seem to know. They did some procedures, they even got a surgeon here to assess. But they are not sure what it is. They are trying different things. I want to be certain about my baby…(Father, 43 years, neonatal sepsis with congenital malformations)

Quote 4: Even when I met the doctor the second time, the day before yesterday, I requested, “Sir, will you confirm again that there will be no problem for the baby later? Can you confirm and tell me if there are any other related organs that will be affected afterwards? Please confirm that there will be no problem”. But the doctor told that it cannot be reassured right now and that we may know as the baby keeps growing. But he said that, “95% she is OK, absolutely OK”…But that once concern will persist in the mind that if there is some problem tomorrow…It is very painful. (Father, 37 years, neonatal sepsis)

Quote 5: When our baby was on ventilator, and regardless of the critical issues, (doctors) should at least initiate mother’s milk. Mother’s milk, everyone knows, is next to amrut [immortality potion]. It is God’s gift. There is no replacement for mother’s milk, even if you give a NASA scientist-prepared milk. (Father, 34 years, neonatal sepsis)

Quote 6: The doctor or sisters did not inform anything. They just took the baby. How should we understand what is happening inside for so long, and why? I am not telling that I doubt the treatment given or anything. They must be treating properly. But they should tell us something. There is no communication at all. What was wrong with the baby? What are they giving the baby? We just sat there outside waiting anxiously. Some lepsis, what is that? (Father, 33 years, neonatal sepsis)

Quote 7: We say ‘Vaidyo Narayano Hari’ (in Sanskrit), meaning ‘Doctor is God’. We have to have faith in them and show our trust. So, we should not question them. Questioning them would be like doubting them, which is incorrect (Father, 37 years, neonatal sepsis)

Box 3 **Finances and culture**

**Financial uncertainty**

Quote 1: No one told us that it would cost so much in this hospital. The previous hospital told us, “If you want the baby to live, go there immediately.” Here every night, I lose my sleep with the tension of by how much will the cost increase if they do additional tests tomorrow. Why don’t people take into consideration that people cannot pay? Had I known this; I would not have agreed to come here. (Grandmother, 47 years, neonatal sepsis, outborn admission)

Quote 2: …It would have [been] very good if transparency was present…they should tell us in advance how much it may cost. If we get an idea about [how much] we have to pay…otherwise where to arrange all of this in time?…On asking, they said, “No, we give you the bill at the last. You pay an advance [money] now”. We wanted to know why we are asked to pay so much and that too within a day? (Father, 28 years, neonatal sepsis with necrotising enterocolitis, outborn admission)

Quote 3: I am not getting correct information. Where is this insurance office and who has to sign? I went outside and looked around everywhere. One person tells me this [Child Insurance X] is applicable only for surgery, not for treatment. But my friend last year claimed it fully for his baby, just for treatment. Not for surgery. I do not know what to do now, this is very confusing. (Father, 30 years, neonatal sepsis, outborn admission)

**Financial constraints**

Quote 4: I could neither attend my father-in-law’s funeral nor travel to avail financial aid, despite being eligible. My wife is unwell. [Nurses] told me I have to be available for my baby. (Father, 33 years, neonatal sepsis, outborn admission)

Quote 5: The other financial assistance is not ready to sanction assistance because the baby’s name is not there on the card. How can we insert the baby’s name when we have not named it? Both doctor and the guruji have advised us to postpone naming ceremony until baby gets OK. We cannot name it till then. (Grandmother, 42 years, neonatal meningitis, outborn admission)

Quote 6: We are paying whatever they ask, whenever they have asked. Health insurance card is there. But it seems it is not applicable for the babies. One more scheme has stopped. He [son in law] does not earn much at all. We sold all our property, we borrowed, we have sold our gold, we have given away our cooking vessels also. From where do we get more now? (Grandmother, 48 years, outborn admission)

**Cultural rituals**

Quote7: So many relatives and elders came to see my baby…I did not let [them] touch him or put ghee on his tongue. But I couldn’t stop them from crowding and burning incense around [baby]. Someone surely gave it [infection] to my baby (tears swelling). (Mother, 27 years, nurse, neonatal sepsis, outborn admission)

Box 4 **Barriers to bonding**

Quote 1: When they brought [me] here, all mothers were with their babies [tears in eyes]. I was the only one staying without my baby here…so painful… [wipes tears] Many mothers without breast milk were keeping their babies [with them]. I had breast milk, but I was unable to keep and feed [my] baby…I felt an uncontrollable rage from within. Many times, I imagined going to the doctor’s room, screaming at [the doctor], secretly taking my baby and running away. [starts crying] (Mother, 21 years, non-local resident)

Quote 2: She has been hysterical…not sleeping, not eating, just crying when she sees others with their babies…She said today “It has been continuously 1 week from that morning [when they took the baby], and I still have not got the baby in my hands. Just give me the baby in my hands, and I will go away where I want to go”. It is such a such a dilemma…We may have to forego the baby or the mother…It is just too painful…She knows baby may not survive if we take it from here. She says that she will take responsibility for whatever happens to the baby. It will not be possible [for us] to stay further. (Grandmother, 48 years, non-local resident)

Quote 3: I could not come here immediately when this happened. I cannot just leave everything and come suddenly. I have to make lot of adjustments at work…I have to work till evening and then I come with home food for my wife. I stay the night and leave in the morning for work. It has been 3 days I have not seen the baby, because visiting hours are closed by the time I can come. Maybe Sunday I can see (Father, 28 years, local resident)

Quote 4:

F: We went to [charity hospital] in the evening as she [wife] was very tired. Doctor said “All water has gone. But the baby’s (foetal) heartbeat is present. It will be difficult to save [the baby] if [the baby] is remains here [at this facility].” The doctor [wife interrupts]

M: There is no facility there, at that place. There is no NICU like this…That is the reason they sent us here saying that (study site) has all the facilities.

F: The doctor checked and said, “Probably water must have leaked. Everything is safe. I will do the C-section in 20 minutes. But it will not be possible to save the baby immediately because it has been a long time since the baby has got infection. To save it” [wife interrupts]

M: [Doctor said] “It will be a 2 hour travel. You will be here and the baby will be there. That will not be correct.” So we came here itself. We came here around 8 o'clock night….delivered around midnight.

(Father (M), 33 years, and Mother (M), 27 years, non-local residents)

Non-local residents were those who stayed in a different town (far away) from that of the study site

### Theme: emotional and financial support during baby’s hospitalisation due to sepsis

Support was in the form of reassurance regarding the baby’s well-being and/or finances (box 5). Participants reported various mechanisms of support that helped them become calm, happy, confident and hopeful during their baby’s hospitalisation (table 3). Mothers typically sought emotional support; fathers sought financial support; and grandparents sought both.

Box 5 **Support**

Quote 1: When I would cry, many doctors and sisters have consoled me saying that nothing would happen [to the baby]. I have seen my husband cry when he is alone. I do not want to stress him more by getting emotional in front of him. (Mother, 21 years, local resident, neonatal meningitis).

Quote 2. I have very good friends, Madam. They keep enquiring about the baby and the situation here. They say- Don’t worry, everything will be ok. Despite their financial problems, they give it to me. I told them I will repay it back once…uh…once I get out of here. So I am not much worried now. (Father, 34 years, non-local resident, neonatal sepsis)

Quote 3: Our minds are not weak like women. We do not need to share what we feel, we do not need to cry. Our duty is to give courage to others, and we have to be strong. If I just sit alone for sometime, I will be OK. (Father, 30 years, local resident, neonatal sepsis)

**Peers**

Quote 4: M: We [mothers] talk. I then understood that many of them have the same problem. In fact, some of the other mothers have more serious problems…Some or the other problem…There are two babies, two months, three months. They have all been admitted.

GM: By seeing them, our pain would reduce. All will be talking only about that. All would be having that same pain. It feels like we are all one. (Mother (M), 21 years and grandmother (GM), 47 years, non-local resident, neonatal sepsis)

Quote 5: They share everything with me… I only share with them what is necessary to be shared… because their mental uh…I mean, capacity is different and our way of thinking is different. There should not be friction. But I welcome if they want to share because [I] will listen if they want to share their pain. Then I will also share [with them]. So I avoid them getting affected because of what I share [with them]. (Father, 34 years, non-local resident, neonatal sepsis)

**Staff**

Quote 6: The sister taught me about the sounds on my baby’s machine [incubator]. I learned to switch it off when it beeps. I feel good and understand it indicates the baby’s temperature. (Mother, 27 years, neonatal sepsis)

**Religion**

Quote 7: [God] is one who saves everybody. One should have that confidence…In times of tension, just pray, meditate. That is our Dharma [duty], Sanatan Dharma [absolute or eternal duty]. Keep doing all your duties, and leave it to God. My Guru tells this to me that God takes care of everyone. We have to keep doing our duty towards living. He [God] is gracing us. We have to surrender to God. (Father, 34 years, neonatal sepsis)

Quote 8: In our side, what we do is something called as “harke” (vow). I did that, saying to God that I would go back and fulfil my vow if the baby becomes well. My mother-in-law had given around 1000 to donate to some place…and additional 200 to give for a puja (prayer). All this has to be done so we don’t leave any stone unturned. (Father, 34 years, neonatal sepsis)

Non-local residents were those who stayed in a different town (far away) from that of the study site

#### Families and friends (quotes 1–3)

Mothers and grandmothers typically sought emotional support, while fathers (breadwinner) sought financial support. Mothers typically confided in their husbands but hesitated when they perceived husbands to be stressed. When husbands were inaccessible because of work, they spoke and frequently cried to their mothers, peers or nurses. Fathers did not actively seek emotional support claiming ‘men had strong minds’, but felt a sense of calm when their friends or family mentioned that ‘all will be well’. Online supplemental file 4 lists the strategies that fathers reported for arranging money.

10.1136/archdischild-2020-319226.supp4 Supplementary data

#### Peers (quotes 4 and 5)

All participants exchanged views with peers on their baby’s condition and unfamiliar NICU environment. Mothers typically connected on their ‘sepsis’ experiences with similar mothers in the NICU (eg, during breast feeding or kangaroo mother care)/dormitory or veteran parents in wards. While fathers sat next to other fathers in the lobby, they exchanged general views about hospitalisation.

#### Staff (quote 6)

Some mothers recalled how nurses, when reached out to, would help in troubleshooting daily problems, and gaining knowledge and competencies. Other mothers reported feeling shy to ask questions to nurses. For some participants, simply observing care coordination, and staff tirelessly ‘do their best despite being busy’ for the baby gave them hope and reassurance.

#### Religion (quotes 7 and 8)

Fathers and grandparents also reported feeling relieved to ‘place their faith and burden on God’ helping them to ‘accept their fate when they cannot do anything’ in this situation. Participants, despite financial constraints, reported offering daily prayers and religious donations (despite financial constraints), and undertaking rituals and sacred vows as directed by priests. Hindu parents reported feeling at ease when priests agreed and encouraged them to delay all rituals until the baby is discharged from the hospital.

## Discussion

We found that dealing with their baby’s sepsis was an unprecedented and shocking emotional and financial experience for families. Our findings corroborate the international qualitative evidence on NICU hospitalisation (including preterm and low birth weight), such as communication gaps, separation from the baby, unexpected changes in parental role, inability to breastfeed, baby’s distress and financial distress.^7 8 35–39^ We add to this by highlighting experiences shaped by the nature of illness, financial, cultural, religious and health service factors relevant to neonatal sepsis.

Our findings reflect those of studies in low-income and middle-income countries (LMICs), where families played a ‘non-participant visitor’ role,^40^ seldom actively seeking information despite their unmet need, except for seeking a guarantee of recovery. Similar to a study on caregivers in Indian intensive care,^41^ these were influenced by an immense faith in doctors, a fear of disturbing staff or being judged for seeking information. Further research should explore if limited parent–staff interactions could additionally be influenced by staff–parent ‘power balance’ in busy LMIC settings.^40 42^ This makes it challenging for families to be eligible to share decision-making with doctors in an unfamiliar and stressful environment.

### Nature of illness

The ‘sudden’ worsening of the apparently healthy baby—a hallmark of sepsis^6^—was a universally present stressor in our study. This was pronounced for babies experiencing severe infections and associated complications, which meant more uncertainty, prolonged stay and increasing costs (eg, due to meningitis, septic shock and comorbidities). Similar to two Scandinavian studies among guardians of critically ill babies, our participants ‘oscillated between hope and hopelessness’,^36^ and experienced constant fear and stress,^7^ making this group ‘extra vulnerable’. This very sudden, unpredictable and life-threatening nature of sepsis is seen as an emotional burden on staff too.^6^ Additionally, similar to our findings, a Swedish study found that this nature of such complex conditions can strain nurse-parent trust.^43^ As recommended by a UK-based study,^44^ ‘tailored’ and ‘multifaceted’ safety netting information should be explored in the Indian context for acute illnesses like child sepsis (eg, use of technology, involving prenatal care for early-onset sepsis, peer support in the absence of family support).

### Financial inclusion

Our study findings reflect those from existing literature on the role of financial constraints.^38^ Families struggled to arrange money at short notice for unpredictable, rising daily expenses. This shock was pronounced for families of cases who were referred without adequate information, were socioeconomically disadvantaged and lacked the resources to avail support. In India, where two-thirds of the population are socioeconomically disadvantaged,^45^ and private level III NICUs offer the costliest neonatal care,^46^ a lack of NICU insurance plans worsens families’ predicament.^47^ Additionally, an American study found that a lack of health insurance for neonatal sepsis can increase mortality and health resource use.^48^ Financing schemes should account for cultural context and financial distress caused by poor referral practices and public healthcare infrastructure.^46^

### Health service barriers

Addressing parent–neonate separation is a core component in FCC. In our study, visiting hours for fathers could be made more flexible. Additionally, hospitalisation of mothers in separate hospitals from their babies hindered bonding for several days. This can be addressed by better planning the continuum of care for mother–baby dyads. Antenatal care providers can minimise mother–baby separation by appropriate childbirth referral practices.^46 49^ Additionally, letting parents periodically see the baby via video connection is a tested approach which can be explored for sepsis.^50^ This can help parents meet their information needs and understand the technology surrounding their fragile baby.

### Cultural practices

Mothers felt powerless to change cultural rituals that may have caused sepsis and which were likely to be repeated again once discharged (eg, prelacteal feeds, burning coal in baby’s proximity).^51 52^ Such practices are frequent in the Indian context, and parents’ decision to avoid them is often superseded by family elders who insist on these practices being conducted.^51^ Strategies to involve and sensitise grandparents and other elders to the dangers of such practices should be explored (eg, video counselling, involving community health centres).^51^

### Support

Familial and religious support was a universal finding in our study. Immediate and extended families provided assistance as needed. Along with religious leaders, families provided emotional support to participants by encouraging faith-based coping, for example, reinforcing faith in God and offering prayers. We could not ascertain if participants questioned their faith or experienced negative religious coping while continuing to observe religious practices.^53^ However, our findings support two studies from HICs,^35 54^ where religion played a positive role among religious parents by providing hope for their baby’s well-being and survival in the wake of uncertainties.

Similar to findings from two studies from HICs, mothers and grandparents bonded over shared experiences with their peers, especially with ‘veteran’ parents who provided emotional support. In realising that there were other babies with sepsis, participants felt less lonely in their NICU experiences and became more hopeful of their baby’s recovery.^55–57^ Additional literature on religious, spiritual and peer support in the Indian context is required (eg, religious spending despite financial constraints).

All our participants were first-time NICU parents and grandparents. Many participants had premature and low-birthweight babies (field notes during clinical rounds). Our interviews were conducted between 2 and 9 days of admission, which may have influenced the experiences. Parents may pass through different stages (novice to expert) between the baby’s admission and discharge in the NICU.^58^ Further research should explore how previous NICU encounters, timing of interviews and prematurity/ low birth weight influence parent stressors in the NICU.

### Strengths and limitations

We believe that this is the first study from an Indian context exploring guardian experiences when dealing with neonatal sepsis while highlighting important illness-related and cultural factors. We did not include staff experiences (to be reported elsewhere) to provide a multifaceted perspective. We did not perform coding in duplicate. Nonetheless, a qualitative expert verified the coding schema and interpretation. Finally, the transferability of findings to other cultural, financial and healthcare contexts should be considered with caution as this study was restricted to a busy private referral level IIIC NICU in coastal South India.

## Conclusion

Designing an FCC for our context requires consideration of stressors that extend beyond the immediate NICU environment and interactions. Understanding the influence of the nature of illness, financial, familial and cultural contexts helps identify the families who are particularly vulnerable to stress. In our context, families, peers and religion were of particular importance for reinforcing the available support system.
