# Supplementary material for: Stressors and support system among parents of neonates hospitalised with systemic infections: qualitative study in South India
Source: Arch Dis Child. 2020 Nov 11;106(1):20–9. doi: 10.1136/archdischild-2020-319226 (PMC7788219; doi:10.1136/archdischild-2020-319226)
Supplement: Supplementary data [file archdischild-2020-319226supp001.pdf]

Supplement 1: Protocol

Table of Contents

Background 2
Objectives 4
Methodology 5
Setting 5
The unit: 5
Population served: 5
Counselling: 5
Families, their availability and accommodation 5
Payment during hospitalization 7
Participants and eligibility 7
Recruitment 7
In-depth interview guide 8
Pilot testing 9
Data collection 10
Data management 11
Data analysis and reporting 11
Ethics and anonymity 13
Rigor 13

Murthy S, *et al. Arch Dis Child* 2020; 106:1–10. doi: 10.1136/archdischild-2020-319226

Background

Neonatal Intensive Care Unit (NICU) hospitalization is traumatic for the neonate and their entire family. Parents of hospitalized premature, low birth weight, critically ill neonates in Level III NICU have reported experiences characterised by stress, insecurity and alienation.[1,2,3,4] A systematic review of qualitative studies highlighted sources of such stress among parents of hospitalized preterm infants: unmet information needs, infant appearance and pain, separation from neonates/ lack of access to neonates.[5],[6]

Neonates of such primary caregivers are at the greatest risk of cognitive and behavioural problems as compared to healthy neonates.[7] Maternal anxiety and depression in the NICU can hinder mother-infant bonding, which subsequently affects the neonate's temperature and heart rate regulation, breathing and crying.[8] For example, perinatal maternal anxiety and depression have seen to detrimentally affect "early cognitive and 12-month communication process", alter "neurosynaptic or regulatory development" and delay developmental milestones.[9] In particular, increased maternal anxiety during 2 to 4 weeks after birth (during hospitalization of a VLBW infant) results in worse fine motor skills at 20 months of age (corrected age).[10]

Sepsis in the baby, however, can pose different challenges for a family compared to a premature baby or a baby with a non-critical illness.[2,11,12] Sepsis is a time-critical condition; a continuum, that can range from a localized infection to severe, life-threatening manifestations including septic shock and cardiopulmonary arrest.[2,12] It's clinical course is frequently unpredictable ranging from non-specific clinical presentation to overt and life-threatening complications. Complications thus can progress suddenly and rapidly leading to multiorgan dysfunction and death, despite corrective measures. The onset and deterioration can be rapid in early-onset neonatal sepsis (EOS). Time-period of EOS varies even within countries, with definitions considering sepsis to occur within 3 days of birth or within 7 days of birth. [13,14] Late-onset sepsis occurs from 3 (or 7) to 28 days of birth. [12,13]

---

[1] Long LE. Stress in families of children with sepsis. *Crit Care Nurs Clin* 2003;**15**:47–53. doi:10.1016/S0899-5885(02)00041-2
[2] Harley A, Latour JM, Schlapbach LJ. The role of parental concerns in the recognition of sepsis in children: a literature review. *Front Pediatr* 2019;**7**:161. doi:10.3389/fped.2019.00161
[3] al Maghaireh DF, Abdullah KL, Chan CM, et al. Systematic review of qualitative studies exploring parental experiences in the Neonatal Intensive Care Unit. J Clin Nurs 2016;25:2745–56. doi:10.1111/jocn.13259
[4] Ireland J, Khashu M, Cescutti-Butler L, et al. Experiences of fathers with babies admitted to neonatal care units: a review of the literature. J Neonatal Nurs 2016;22:171-6. doi: 10.1016/j.jnn.2016.01.006
[5] Obeidat HM, Bond EA, Callister LC. The parental experience of having an infant in the newborn intensive care unit. The Journal of perinatal education. 2009 Jan 1;18(3):23-9. https://www.ncbi.nlm.nih.gov/pmc/articles/PMC2730907/#bib16
[6] Dutta S, Mahajan R, Agrawal SK, Nehra R, Narang A. Stress in fathers of premature newborns admitted in a neonatal intensive care unit. Indian pediatrics. 2016 Apr 1;53(4):311-3. https://indianpediatrics.net/apr2016/apr-311-313.htm
[7] Sanders MR, Hall SL. Trauma-informed care in the newborn intensive care unit: promoting safety, security and connectedness. J Perinatol 2018;38(1):3-10. Doi: 10.1038/jp.2017.124
[8] Klawetter S, Greenfield JC, Speer SR, Brown K, Hwang SS. An integrative review: maternal engagement in the neonatal intensive care unit and health outcomes for US-born preterm infants and their parents. AIMS Public Health. 2019;6(2):160.https://www.aimspress.com/fileOther/PDF/aimsph/publichealth-06-02-160.pdf
[9] Hoffman C, Dunn DM, Njoroge WF. Impact of postpartum mental illness upon infant development. Current psychiatry reports. 2017 Dec 1;19(12):100. https://link.springer.com/article/10.1007/s11920-017-0857-8
[10] Greene MM, Rossman B, Meier P, Patra K. Elevated maternal anxiety in the NICU predicts worse fine motor outcome in VLBW infants. Early human development. 2018;116:33-9. DOI: 10.1016/j.earlhumdev.2017.10.008
[11] Singh M, Gray CP. Neonatal Sepsis. [Updated 2020 Jun 2]. In: StatPearls [Internet]. Treasure Island (FL): StatPearls Publishing; 2020 Jan-. Available from: https://www.ncbi.nlm.nih.gov/books/NBK531478/
[12] Rubarth LB. The Lived Experience of Nurses Caring for Newborns With Sepsis. Journal of Obstetric, Gynecologic & Neonatal Nursing. 2013. 32(3), 348–356. doi:10.1177/0884217503253437
[13] Murthy S, Godinho MA, Guddattu V, Lewis LE, Nair NS. Risk factors of neonatal sepsis in India: A systematic review and meta-analysis. PloS one. 2019 Apr 25;14(4):e0215683.
[14] Cortese F, Scicchitano P, Gesualdo M, Filaninno A, De Giorgi E, Schettini F, Laforgia N, Ciccone MM. Early and late infections in newborns: where do we stand? A review. Pediatrics & Neonatology. 2016 Aug 1;57(4):265-73.

The experience can be frightening circumstance for parents.11 The stressors involved can be multiple and affect different fronts of lives of families experience a hospitalization for their baby's sepsis.[2] Qualitative studies from HICs suggests that parents of such acute illness/ critically ill infants have been said to be in constant fear, undergo a "shock phase", and face more stress and ill health than counterparts with non-critically ill infants.[15] However, there is scarce and inconclusive evidence on the nature of the neonate's illness influencing parent stress in the NICU, particularly from LMICs including India.

Qualitative evidence from India describing the psychosocial needs of parents of sick neonates from India is scarce. One aimed at 'identifying gaps and challenges post-FCC implementation' by understanding the providers' and families' perceptions and experiences from a government tertiary care NICU.[16,17] In-depth interviews with 12 parents revealed that parents adapted better to the NICU environment, despite 'infrastructural stress factors' with improved understanding of the care provided, improved competencies, and access to their baby (brought about by the FCC).[16] Another study focusing on maternity care in government secondary healthcare facility study used 24 in-depth interviews with postpartum mothers to understand the user experiences regarding quality of maternal health care services, and focused on intrapartum challenges.[17]

Studies have numerically 'scored' parent's experiences using validated quantitative scales to measure NICU-related stress. In such studies, infant behaviour, alteration in parental role, neglect of home affairs, and staff interactions were stressors, of which the first three have consistently received high stress scores.[6,19] Another study on fathers of hospitalized preterm low birth weight neonates in a public Level III neonatal unit found financial burden to be a significant stressor. Baby's condition, socio-demographics, father's age, family history, understanding technology surrounding the child, were suggested to be influence parent stress levels. [6,18,19]However, a study on 343 parents of neonates admitted for at least 48 hours reported found no significant influence of the nature of the illness or requirement of therapy (e.g. ventilation-required for systemic infections, depending on the type and severity) on parent stress levels. [19] Such quantitative studies can do little to capture in-depth experiences of familial needs and contexts in the Indian context.[19]

Coping for such families has been facilitated by emotional and professional support, parent education and facilitation, information support.[5,6] Understanding families' cultural contexts (e.g. religious and daily life practices) is essential for effectively utilizing their capabilities in designing and supporting FCC. The staff need to help families of septic hospitalized babies

---

[15] Karlsson C. Health promotion work: Pediatric nurses perspectives on the needs of supporting parents with critically or chronically ill chidren: A qualitative interview study of Sweden

[16] Sarin E, Maria A. Acceptability of a family-centered newborn care model among providers and receivers of care in a Public Health Setting: a qualitative study from India. BMC health services research. 2019 Dec;19(1):1-1.https://www.ncbi.nlm.nih.gov/pmc/articles/PMC6427855/

[17] Bhattacharyya S, Issac A, Rajbangshi P, Srivastava A, Avan BI. "Neither we are satisfied nor they"-users and provider's perspective: a qualitative study of maternity care in secondary level public health facilities, Uttar Pradesh, India. BMC health services research. 2015 Jun;15(1):1-3.https://www.ncbi.nlm.nih.gov/pmc/articles/PMC4584124/

[18] Hall EO. Being in an alien world: Danish parents' lived experiences when a newborn or small child is critically ill. Scandinavian journal of caring sciences. 2005 Sep;19(3):179-85. Doi: 10.1111/j.1471-6712.2005.00352.x

[19] Varghese M. A study on parental stress in the neonatal ICU using parental stressor. Pediatrics. 2015 Feb 1;135(Supplement 1):S9. https://pediatrics.aappublications.org/content/135/Supplement_1/S9.1. DOI: 10.1542/peds.2014-3330O

effectively handle their stress by understanding their context and coping.[5,20,21] Thus, understanding support systems may prove helpful in understanding parental needs during this stressful time.[21]

India witnesses the highest burden of clinical neonatal sepsis in the world.[22] This condition is additionally the leading cause of morbidity and deaths in LMICs.[11,23] Despite policy recommendations on introducing FCC in public healthcare, progress has been slow in India.[24,25] Neonatal care continues to be technology-driven and provider-centered, with limited parent involvement.25 Research in India exploring the psychosocial needs of parents of hospitalized neonates is scarce, with none focusing on sepsis.

Aim: This exploratory study therefore aims to explore the experiences of parents and accompanying attendants, having a baby admitted for the treatment of sepsis, at a Level IIIc NICU in coastal South India. The study is anticipated to provide baseline inputs to staff and hospital administrators regarding guardian experiences in the setting. This will inform the design of need-based strategies to improve their NICU experiences and satisfaction (based on the domains found from our study). Our findings may additionally inform the need and subsequent design of a larger mixed-methods intervention study on family-centered care in our setting.

Objectives

Specifically, the objectives of this study, conducted in coastal South India, are to understand the:

1. stressors for parents and accompanying attendants when they have a baby admitted for the treatment of sepsis in a private tertiary care Level III C NICU
2. sources of support for parents and accompanying attendants when they have a baby admitted for the treatment of sepsis in a private tertiary care Level III C NICU

This qualitative study is part of a larger doctoral research using multi-methods to understand the prevention and management of neonatal sepsis in a coastal district in South India. The topic for the research was inspired by and an offshoot of a mixed-methods study titled "Determinants of neonatal pneumonia and the factors associated with mortality of neonatal pneumonia: a systematic review combined with qualitative research approach", for which SM worked as a research associate. The research design included a cost-of-illness followed by a qualitative study. The qualitative study was planned as an exploratory study to understand the experiences of parents and accompanying attendants of neonates admitted in the NICU with sepsis. This study was thus meant to provide an understanding of why families seek newborn care at a private institution (separate paper), and what their experiences were (in terms of

[20] Kokorelias KM, Gignac MAM, Naglie G, et al. Towards a universal model of family centered care: A scoping review. BMC Health Serv Res 2019;19:564. doi:10.1186/s12913-019-4394-5

[21] Hua A, Pham T, Spinazzola R, et al. Support systems for NICU parents [abstract]. Pediatrics 2018;141(1 MeetingAbstract):561–561. doi:10.1542/PEDS.141.1_MEETINGABSTRACT.561

[22] Panigrahi P, Chandel DS, Hansen NI, *et al.* Neonatal sepsis in rural India: timing, microbiology and antibiotic resistance in a population-based prospective study in the community setting. *J Perinatol* 2017;**37**:911–21. doi:10.1038/jp.2017.67

[23] Seale AC, Blencowe H, Manu AA, Nair H, Bahl R, Qazi SA, Zaidi AK, Berkley JA, Cousens SN, Lawn JE., pSBI Investigator Group. Estimates of possible severe bacterial infection in neonates in sub-Saharan Africa, south Asia, and Latin America for 2012: a systematic review and meta-analysis. Lancet Infect Dis. 2014 Aug;14(8):731-741.

[24] Verma A, Maria A, Pandey RM, et al. Family-centered care to complement care of sick newborns: A randomized controlled trial. Indian Pediatr 2017;54:455–9. doi:10.1007/s13312-017-1047-9

[25] Maria A, Dasgupta R. Family-centered care for sick newborns: A thumbnail view. Indian J Community Med 2016;41:11–5. doi:10.4103/0970-0218.170957

stressors and support system- addressed in this paper) in this setting, in the context of the doctoral research topic of neonatal sepsis.

Methodology

We will conduct a qualitative study, using principles of grounded theory,[26,27] in a tertiary care teaching hospital in Udupi, a coastal district in South India, based on methodological aspects previously described elsewhere.[28]

Setting

We provide a detailed reporting of the setting our study context to understand the transferability of our findings, and add to the rigor of our study.

The unit: The neonatology unit houses a reception-cum-waiting area, NICU (Level III C), a stepdown nursery, a septic ward, a breastfeeding room and a discharge ward (mother-neonate dyads stay together around the time of discharge)- all on the same floor.

Population served: The admissions vary according to type of neonatal units in India. One fifth of all newborn possibly requiring rural SNCU (Level II),[29] with an estimated 1.2 million neonates admitted to 525 SNCUs in India.[30] Another study reported the following magnitude of neonatal admissions based on sector and level of care: Government secondary 91 (55-138 per month), government medical college 143 (110-176), private medical college 31 (21-45), private tertiary care 47 (38-53).[30,31]

As per the treating clinician's estimate, roughly over 120 neonates are admitted to the unit per month, of which five to eight cases belong to sepsis. This busy unit, utilized by both urban and rural population belonging to all socioeconomic strata, serves as the referral center for pediatric and neonatal care for eight surrounding districts. The total population of these districts is nearly 0.5 million, of which children aged 0-6 years comprise nearly 0.7 million[32] The unit receives both inborn and outborn neonates.

Counselling: Once a neonate is admitted to the unit, parents and accompanying attendants of all hospitalized neonates are mandated to undergo counseling by a consultant (neonatologist) in the presence of the one to two on-call postgraduate(s). On an average, nearly 45 families undergo counseling every day. Each counseling session lasts between 15 to 20 minutes and is provided in the language comfortable to the families. Families are free and encourage to voice their queries during the counseling.

Families, their availability and accommodation: These comprise of mother, grandparents, fathers, other family members (e.g. brothers); either together or individually. Approximately 45

[26] Charmaz K. Constructing grounded theory: A practical guide through qualitative analysis. Sage; 2006 Jan 13.

[27] Hennink M, Hutter I, Bailey A. Qualitative research methods. SAGE Publications Limited.

[28] Nair NS, Lewis LE, Lakiang T, Godinho MA, Murthy S, Venkatesh BT. Risk factors and barriers to case management of neonatal pneumonia: protocol for a pan-India qualitative study of stakeholder perceptions. BMJ Open. 2017;7(9):e017403. doi:10.1136/bmjopen-2017-017403

[29] Neogi SB, Malhotra S, Zodpey S, Mohan P. Assessment of special care newborn units in India. Journal of health, population, and nutrition. 2011 Oct;29(5):500.https://www.ncbi.nlm.nih.gov/pmc/articles/PMC3225112/

[30] Hanson C, Singh S, Zamboni K, Tyagi M, Chamarty S, Shukla R, Schellenberg J. Care practices and neonatal survival in 52 neonatal intensive care units in Telangana and Andhra Pradesh, India: A cross-sectional study. PLoS medicine. 2019 Jul 23;16(7):e1002860.https://journals.plos.org/plosmedicine/article?id=10.1371/journal.pmed.1002860

[31] Karambelkar G, Malwade S, Karambelkar R. Cost analysis of healthcare in a private sector neonatal intensive care unit in India. Indian Pediatr. 2016 Sep 1;53(9):793-5. https://www.indianpediatrics.net/sep2016/793.pdf

[32] https://www.census2011.co.in/

to 48% of families reside in towns outside that of the study site. Such families choose to stay at an accommodation (a ‘dormitory’ opposite to the hospital) provided by the hospital for the duration that their baby is hospitalized. Mothers of inborn babies are usually admitted in wards one to two floors above or a floor below the neonatal unit until their post-delivery recovery is complete (anytime between 3 days to few weeks). Families (including the mother) can choose to stay in the dormitory.

Mothers do not have limitation on the ‘visiting hours’ and are allowed inside the NICU whenever they wish. Attendants other than mothers are allowed inside the NICU only during the visiting hours. Mothers are allowed to breastfeed every two hours, provide kangaroo mother care (KMC) and bond with their baby (depending on the clinical condition of the baby, adequacy of breast milk, and physical condition of the mother to breast feed) in the NICU. Mothers sit on a chair next to the baby’s bed while breastfeeding and providing KMC. They usually smile and talk to neighbouring mothers (when present) involved in similar activities for their babies.

Fathers, grandmothers and others usually helped the mother by bringing food and beverages (from home or canteen), medicines, clothes or anything that was needed for her. They additionally help pump her breast milk, and transport it (every two hours) to the staff for the baby. This was important for babies whose could not be breast fed directly due to the type and severity of the systemic infection, and thus their clinical condition. Families also helped when the staff required them for the baby.

Mothers: First-time mothers followed the practice of spending the few months of before and after childbirth at their maiden homes. Hence grandmothers typically accompanied the mothers for pre-delivery hospital visits, during and after childbirth. It was also possible that mothers were absent from the study site, during their baby’s hospitalization, for a few days to a fortnight. Reasons for this included that the mother had delivered at the referring facility and had not completely recovered when the baby had to be referred to the study site. In such cases, only grandmothers would be available at the NICU as the baby’s father would be away at work.

Fathers: Fathers typically would be available during lunch time, evening and night, as they were away at work during the day. Some fathers would not be available, for weeks, as they were employed in different Indian districts/states, or country. Many fathers (except daily wagers) come on alternate days during the weekdays (Monday to Saturday).

Grandparents: The mother’s mother typically accompanies the mother at the study site during the baby’s hospitalization, also reported in literature from our context.[16] Working grandmothers staying in the proximity of the study site, are available part-time with the mother. Such grandmothers also bring home-cooked food on a daily-basis for the mother, and return to their residence during the night. Non-working grandmothers, not staying in the vicinity, stay with the mothers in the dormitory between days to weeks, depending on their responsibilities at their homes. Some grandparents would be available next to the mothers full-time in the wards as well. Maternal fathers and paternal parents rarely stay with the mothers or fathers during the hospitalization, and might visit on a weekend.

Others: Occasionally, the paternal brother or uncle, or maternal sister represents the parent for a day or during counseling during the baby's hospitalization, typically when the father was unable to visit.

Payment during hospitalization: Costs typically comprise: baby's hospitalization; accommodation, food, clothing, transport and other (religious activities) costs for the families. The costs are borne partially or completely by the families, depending on availability and utilization of health insurance (private or public) and other local financial assistance schemes. Limited financial assistance for certain segments of the population (e.g. below poverty line, religious minorities) are provided by charities/trusts, based on official recommendation by the unit. The coverage and type of payment mechanisms of health insurance varies by the provider. For example, the insurance may pay the provider directly eliminating the need for families to pay during the hospitalization (as opposed to reimbursement of expenses paid by the families, and subject to submission of bills). Out-of-pocket expenses (e.g. transport) were not covered under any health insurance in this setting.

Participants and eligibility

Participants will include parents or grandparents of neonates (0-28 days) admitted to the NICU for the management of one or more systemic infections. The systemic infections in this study could include one or more of the following: septicemia, pneumonia, meningitis, osteomyelitis, arthritis and urinary tract infections.[33] A purposive sampling will be used to include parents and accompanying attendants immediately or within the first few days after diagnosis. This was done to capture and understand their experiences as soon as they knew of their baby's condition. Thus, some apriori exclusions are (i) a parent or the spouse who was unavailable at the study site during the data collection period, and (ii) parents with babies who are in the midst of treatment (e.g. meningitis which requires 21 days of treatment) or closer to discharge/ have been discharged from the unit). Additionally, parents and accompanying attendants who refuse to provide written informed consent will be excluded from the study. Both early- and late- onset sepsis cases were included. No other exclusion criteria were applied.

Recruitment

Recruitment of the parents and accompanying attendants will be done on a rolling basis (prospectively, as admissions occur), at the study site, within the first two days of admission with a diagnosis of a systemic infection. SM will visit the neonatology unit Monday through Saturday and collect the list of babies admitted in the NICU every day. This means that, at the time of recruitment, the baby was admitted and would have just started receiving treatment for the infection in the unit. For days which were missed due to SM's absence (e.g. sickness, some Sundays), this list would be updated on the subsequent day. SM will subsequently make a list of babies diagnosed with a systemic infection by consulting the staff and reviewing the baby's medical charts each day. The baby is typically identified in the unit as "Baby of <Mother's name>" in the records, unless the family has already named the baby.

Parents and accompanying attendants will be approached when they are seated in the waiting lobby, by telephone or personal visits to the hospital dormitory where they reside. SM will identify and approach the parents/ accompanying attendant responding to this identifier in the

[33] Sankar MJ, Agarwal R, Deorari AK, Paul VK. Sepsis in the newborn. Ind J Pediatr. 2008;75(3):261–266

reception area, between 10 am to 5.30 pm. For mothers admitted in the ward (See “Setting” above), SM approached the mother in the ward, taking assistance of the nursing staff to identify the mother. Additionally, SM will take assistance of the unit staff to identify a particular parent/ accompanying attendant was present inside the unit. This was deemed necessary; in case the parents/ accompanying attendant would be unavailable in the reception area or the wards. SM will aim to identify and approach the parents/ accompanying attendant on the same or next day of diagnosis of sepsis. If this was not possible (e.g. absence of SM, diagnosis made between 5.30 pm to midnight, unable to locate parents/ accompanying attendant), an attempt will be made on the subsequent day.

Once the parents and accompanying attendants are approached, SM will brief them in a private room beside the reception area. They will be invited to participate by providing a participant information sheet and informed consent form in the language preferred by the parent(s). SM will explain the contents of these documents in a language preferred by the participants (e.g. about the voluntary nature of the research study and right to withdraw at any point; need, aim and methods of research; why they are being invited; what data will be collected and how; anticipated harms and possible benefits to the participant, and dissemination modalities). The details of the researcher (e.g. present position of the researcher, objective of this doctoral research study and how it ties to the larger career aims of the researcher) will also be explained.

If the spouse/parent is unavailable, SM will enquire regarding their availability in the hospital to invite them for the study. The parents/ accompanying attendant was requested to provide the spouse/ parent’s phone number for subsequent contact. Details of when the spouse or parent would be subsequently available will be noted for subsequent contact (if contact details were not provided). Parents and accompanying attendants will also be requested to provide their contact number and/or their location in the hospital (e.g. room number in dormitory) to contact them after one to two days for their decision to participate. If they refuse to provide this information, they will be informed that SM would approach them in the unit after the counseling, after one to two days, for their decision. Parents and grandparents will be requested to participate individually for the interview.

The same process will be followed to recruit the spouse/parent on subsequent contact. If they agreed to participate, SM will take written informed consent in duplicate and subsequently recruit the participant. After recruitment, participants will be requested to provide a date and time for interview.

In-depth interview guide

A semi-structured IDI guide will be used to interview participants. We recognize that there is a scarcity of qualitative studies exploring the experiences of parents’ experience in the NICU, particularly in the context of illnesses like sepsis. Thus, we intended to keep the questions broad and allow the participant to freely express themselves. Qualitative literature review informed our IDI guide in understanding the concepts, developing questions and probes. In addition, consultation with experts and revised following a pilot study on 5 parents (not included in the main study) informed the final IDI guide. The analysis from the pilot study will inform (i) the refinement of the IDI guide, and (ii) the development of deductive codes for the two deductive themes (described in “Data analysis” below). The final IDI guide had the following domains: participant background, experience of baby in the NICU, stressors, positive

and negative emotions, what their baby may go through, their coping mechanism, who they discuss their experiences with, prior experience with sick baby, finances for NICU-related costs, financial impact on family and current financial coping, perceived support that they wished to have received. Participants will be encouraged to describe their feelings or emotions they experience during a stressor and support.

Pilot testing

Three fathers and two mothers were recruited for pilot testing, which served to revise the IDI guide. In addition to modifying probes and the language/structure of questions, it modified the following aspects of the study:

- Challenges in operationalising the conceptual framework: We planned to use the concepts from the Transactional Model of Stress and Coping as a guiding framework for data collection and analysis. However, we found operational challenges during the pilot study, which made it extremely difficult to operationalize this model as recommended by the "Transactional Model of Stress and Coping" (TMSC) to develop the IDI guide.[34] For example, we were unable to administer the "Ways of Coping Questionnaire" (an essential component of the model to understand coping mechanisms) due to contextual challenges. For example, mothers were repeatedly reluctant to respond to the questionnaire in addition to giving an interview citing health-related issues after childbirth. Many fathers simply lacked the time to participate in interview, as they juggled between tasks of delivering food to the wife and arranging to pay for NICU-related costs. Participants during the pilot study thus simply refused to accept the questionnaire. We had to thus modify our methodology to fit our research inquiry and study context, and proceed without using the conceptual framework. We finally decided to explore the concepts of stress and support system. Further, we will attempt to capture the factors that enable parents and accompanying attendants to adapt to the neonatal environment, and those which make the stay more difficult.[12,35]

About TMSC: Since our interest was to study the stress and coping among parents during their baby's hospitalization, we intended to use TMSC as it evaluates the process of stress and coping during important life events. TMSC assesses how people appraise a situation as being stressful (in the form of emotions, thoughts, behaviour) and subsequently judge their ability to cope (demands versus resources) if the event is found stressful. TMSC measures and explores coping strategies through a "Ways of Coping" Questionnaire. The original version is a 66-item checklist with responses to be scored on a four-point Likert scale, denoting the extent to which the listed strategies were used/ not used in a particular situation considered stressful. The model further also suggests that certain people are particularly more vulnerable to 'stressful' events, because of their background, environment and/or personality.[34,36]

- Including grandparents as participants: Our initial eligibility criteria included only parents. This posed challenges during the pilot study as grandparents were frequently present with mothers in the unit. Mothers refused to be interviewed without the presence of their mothers, and their mothers also responded to the questions (despite

[34] Lazarus RS, Folkman S. Stress, appraisal, and coping. Springer publishing company. 1984
[35] Hall SL, Hynan MT, Phillips R, Lassen S, Craig JW, Goyer E, Hatfield RF, Cohen H. The neonatal intensive parenting unit: an introduction. Journal of Perinatology. 2017 Dec;37(12):1259.
[36] Folkman S. Ways of coping checklist (WCCL). Encyclopedia of behavioral medicine. 2013:2041-2. https://link.springer.com/referenceworkentry/10.1007%2F978-1-4419-1005-9_222

briefing them not to, before the interview began). The mother was also emotional and mentioned that she felt more confident to answer in the presence of her mother. Thus, we considered including the grandparents in such circumstances, as they were equally (and sometimes more) involved as the parent, in the activities in NICU.

- An explicit finance-related question: We had included 'finance' as a probe in the initial version of IDI guide. We found, during pilot testing, that participants spoke about the different mechanisms of arranging money, which was a struggle in itself and stressful. Thus, an explicit finance-related question with tick boxes served as a guideline for the interviewer to go deeper according to the option mentioned by the participant (ticked by the interviewer).

- Recruitment and interviewing: Participants were also requested on feedback if they were comfortable with the way they were approached during recruitment. We also learned that fathers might decide for their wife's participation in the study, or can insist that they themselves participate in their wife's stead and speak for their wife.

- Participant checking and feedback: We were unable to perform participant checking for other interviews due to contextual challenges. For example, participants repeatedly cited a lack of time, ill-health or being too worried to give time after interviews. Additionally, we were unable to meet many fathers again during the data collection timings.

Data collection

Data will be collected using face-to-face in-depth interviews. Participants will be contacted telephonically or in-person (when available) on their preferred date and time of interview. Attempts will be made to include both the mother and father, and to interview them separately (i.e. not in each other's presence) in order to avoid one influencing the other's views. If the parents are unwilling for this arrangement, data will be collected from them seated together. Interviews will be conducted in a language (English, Kannada, Tamil, Marathi, Hindi), location and time preferred by the participants. Additionally, observations (in the form of field notes) during clinical rounds and patient counseling will be made to understand the baby's clinical condition and progress, the participant- baby and participant- staff interactions in the NICU environment. Observations of patient counseling included the regularity of the participant to counseling sessions, and the number and relation of people accompanying/ substituting the participant for the counseling. This information was collected from the unit staff and registers after each patient counseling session. Multiple sources of data collection will thus help us understand if this converges or diverges with the participants' responses regarding these aspects during the interview.

Privacy will be ensured by interviewing the participants only in the presence of the researcher. Participants will be contacted for repeat interviews in instances where a deeper understanding of concepts/ clarification of concepts is required after the initial interview. Data will be collected until information saturation is reached and no new meaningful concepts emerge from subsequent interviews. [26,37]

[37] Saunders B, Sim J, Kingstone T, Baker S, Waterfield J, Bartlam B, Burroughs H, Jinks C. Saturation in qualitative research: exploring its conceptualization and operationalization. Quality & quantity. 2018 Jul 1;52(4):1893-907.https://www.ncbi.nlm.nih.gov/pmc/articles/PMC5993836/

Audio-recording, with prior permission from the participant, of the interviews will be done on an electronic voice recorder (Sony ICD-PX440). Interviews will be hand-written for participants who deny permission for audio-recording. The recordings will be transferred and stored on a computer for subsequent transcription. Field notes will be recorded both during and after the interviews to record: concepts or keywords that emerge from the interview, participant non-verbal cues and post-interview reflections (e.g. participant hesitations; interaction and behaviour between the couple, and individually, during couple interview; codes which need further exploration). Audio recording of the interviews will be done to avoid missing information, to retain participant narratives as is said by the participants themselves, and to focus on the expressions in the text, and minimize researcher interpretations. For hand-written interviews, the notes made during the interview will be read out to the participant immediately after the interview to verify the information. These will add to the dependability of the study.[38]

We will maintain a detailed report of the research plan, its stage-wise implementation, challenges faced and strategies instituted. Additionally, a thick description of the results will be first written up, from which the manuscript will be prepared. These will be shared for external review with research team members. The researcher was adequately trained in qualitative research and analysis, and mock interviews conducted- all of which helped to be constantly mindful of subjective interpretations to minimize its influence on the data. This will be done to ensure that interpretations are derived from data to add to the rigor in terms of confirmability of the findings.[41]

Data management

The IDIs will be transcribed (SM) verbatim, translated, and supplemented with field notes, if and when necessary. Hand-written observations and field notes will be typed, and will undergo the same cleaning and analysis process as IDI transcripts. Transcripts and field notes will be cleaned only to anonymize and remove any potential identifiers.[39] Signed informed consent sheets will be stored in a separate physical cabinet and will not be linked to the unique code. Anonymized transcripts, field notes and analysis files will be stored separately on a password-protected cloud server.

Data analysis and reporting

ATLAS.ti software will be used to facilitate the management and analysis of qualitative data. A thematic analysis will be performed, as recommended in literature.26 We will derive our sub-themes conceptually (instead of from an existing theoretical framework) by closely examining our interview transcript data, based on the patterns and ideas that repeatedly emerged from our data.26 Interviews will be transcribed, and field notes will be typed following each interview. This will be followed by repeated listening of the interview, and reading and re-reading of the transcripts and field notes to get an understanding of the entire experience described by the participants. Immersion in the data will thus help in rigor by ensuring the credibility of our findings. Coding of the data will be done (explained later in this section). Data will be organized to list the stressors and support systems mentioned by the participant. Emotions associated with the stressor and support system will be listed. Observations from

[38] Korstjens I, Moser A. Series: Practical guidance to qualitative research. Part 4: Trustworthiness and publishing. European Journal of General Practice. 2018 Jan 1;24(1):120-4. https://www.tandfonline.com/doi/pdf/10.1080/13814788.2017.1375092

[39] Kaiser K. Protecting respondent confidentiality in qualitative research. Qual Health Res 2009;19:1632–41.doi:10.1177/1049732309350879

field notes will be explored during subsequent interviews (e.g. patterns or repetition of emotions, and while describing a particular stressor or support system; similarities and differences during couple interviews). These concepts/ phrases will guide the further exploration in the subsequent interviews until no new concepts emerge and data saturation is attained.[28,40] This will also allow guide in actively identifying disconfirming sources for data that "does not fit" or different from that which repeatedly occurs, to aid in the credibility of our study.[38]

Two cycles of coding will be conducted as recommended in literature.[26] The first cycle will provide a list of codes which will be closer to the data. Codes could range from a single word to a phrase to a short statement. We will list inductive (emerging from data) codes, in addition to codes already identified (IDI guide, identified during pilot study), until we achieve data saturation. A code book will be developed by SM under the guidance of HH and AB. The second cycle will generate code families or categories, by grouping similar codes in a meaningful way. Subsequent grouping and categorization will be under two themes to address our research questions. A second qualitative researcher/expert (HH) will check the coding cycles, referring to the IDI guide to add to the confirmability of the study. The coders will discuss the coding schemes and reach consensus. Codes will be renamed as deemed appropriate.

Stressors will be coded by identifying those factors that the participant describes as making them feel strained, tensed, distressed, or something which avoided/did not prefer. Descriptions, by participants, of what supported them/ helped them feel better or cope/reduce their stress, and how, will be analyzed under the deductive theme of "Support System". Participants may report more than one feeling or emotion for a stressor/ support system (found from pilot-study). This will be captured during the analysis and reported in text and tabular formats. Interpretation will be done by the interviewer (SM), two qualitative research experts (AB, HH) with inputs from a neonatologist (LL). The findings will be shared with research team colleagues and doctoral advisory committee members for external review to ensure confirmability and credibility of the findings.[38,41]

Data will be presented in textual and tabular (e.g. participant characteristics and background) formats. Select participants' quotations will be provided throughout the text to support the interpretation of the results. Non-verbal cues from field notes will be reported (where present) in the participant quotations in parenthesis and in the overall description of results/ sub-themes. In addition, the limitation in terms of context will be provided, all of which will add to rigor in terms of understanding the transferability of findings.[38] Reporting of the study will be informed by "The Standards for Reporting Qualitative Research" (SRQR)[42] and the "Consolidated criteria for reporting qualitative research" (COREQ).[43]

[40] Saunders B, Sim J, Kingstone T, Baker S, Waterfield J, Bartlam B, Burroughs H, Jinks C. Saturation in qualitative research: exploring its conceptualization and operationalization. Quality & quantity. 2018 Jul 1;52(4):1893-907. https://www.ncbi.nlm.nih.gov/pmc/articles/PMC5993836/

[41] Mirlashari J, Brown H, Fomani FK, de Salaberry J, Zadeh TK, Khoshkhou F. The challenges of implementing family-centered care in NICU from the perspectives of physicians and nurses. Journal of pediatric nursing. 2020 Jan 1;50:e91-8. https://www.pediatricnursing.org/article/S0882-5963(18)30402-0/fulltext#back-bb0160

[42] O'Brien BC, Harris IB, Beckman TJ, Reed DA, Cook DA. Standards for reporting qualitative research: a synthesis of recommendations. Academic Medicine. 2014 Sep 1;89(9):1245-51.

[43] Tong A, Sainsbury P, Craig J. Consolidated criteria for reporting qualitative research (COREQ): a 32-item checklist for interviews and focus groups. International journal for quality in health care. 2007 Dec 1;19(6):349-57.

Ethics and anonymity

Principles in the Declaration of Helsinki will be followed. A scientific committee approved the study protocol. Administrative permissions were obtained from the medical superintendent of the hospital, and the head of the neonatology unit. Subsequently, ethical clearance was provided by the Institutional Ethics Committee of the hospital. Privacy and confidentiality of participants will be maintained throughout the study. Each participant will be provided with a "unique identification code" identifying the type of participant (e.g. father, mother, grandmother). Transcripts will be cleaned and anonymized to remove any identifiers. Participants will continue to remain unidentifiable in all study reports.

Rigor

Trustworthiness of our data will be ensured by instituting measures for credibility, confirmability, dependability, transferability and positionality.[44,45]

Credibility and confirmability: Multiple sources of data collection (participant accounts, field notes from observation of interactions) will help us understand and triangulate the data from participants' responses. Audio recording of the interviews will be done to avoid missing information, to retain participant narratives as is said by the participants themselves, and to focus on the expressions in the text, and minimize researcher interpretations. For hand-written interviews, the notes made during the interview will be read out to the participant immediately after the interview to verify the information. SM has been adequately trained in the methods and analysis of qualitative research, and has previously conducted 63 IDIs for a pan-India qualitative study on neonatal health. These will help to be constantly mindful of subjective interpretations to minimize its influence on the data, and that interpretations are derived from data. A second qualitative expert (HH) will check the coding cycles. Interpretation will be done by the interviewer (SM), two qualitative research experts (AB, HH) with inputs from a neonatologist (LL). We will continuously immerse in the data (active and repeated listening of interviews, reading and re-reading of interview transcripts). The findings will be shared with the research team colleagues and doctoral advisory committee members for external review to maximize confirmability and credibility of the findings.

Dependability: We will maintain a detailed report of the research plan, its stage-wise implementation, challenges faced and strategies instituted. Additionally, a thick description of the results will be first written up, from which the manuscript will be prepared. Description of analysis, direct quotations and emotions described by the participants will be provided.

Transferability: An explicit protocol has been developed and reported here, which helps in understanding the study design and context. The study's limitation in terms of context will be provided in the manuscript.

Positionality: The interviewer (SM) is an unmarried female PhD student (in the third year at the time of interviewing), who was external to the NICU and not a member of the clinical NICU team. She has formal qualifications in dentistry (BDS) and public health (MPH), and has research experience since 2011. SM is not related to, and is not a part of, the neonatology unit where the study was conducted.

44 Korstjens I, Moser A. Practical guidance to qualitative research. Part 4: trustworthiness and publishing. Eur J Gen Pract 2018;24:120–4. doi:10.1080/13814788.2017.1375092
45 Lincoln YS, Guba EG. Naturalistic inquiry. New Delhi: SAGE Publications India Pvt Ltd 1985
