# Supplementary material for: Stressors and support system among parents of neonates hospitalised with systemic infections: qualitative study in South India
Source: Arch Dis Child. 2020 Nov 11;106(1):20–9. doi: 10.1136/archdischild-2020-319226 (PMC7788219; doi:10.1136/archdischild-2020-319226)
Supplement: Supplementary data [file archdischild-2020-319226supp002.pdf]

## Supplement 2: In-depth Interview Guide

**1. Let us talk about your baby. Can you tell me why you are here?**
*Probe: present condition, knowledge about diagnosis*

**2. Talking about your experience, how do you feel about having your baby admitted here?**
*Probe: different feelings- positive and negative, spouse's feelings*

**3. What stresses you about having your baby admitted here?**
*Probe: baby, environment, peers, staff*

**Alternate question if the participant doesn't respond: Can you tell me an instance when you felt negative (e.g. anxious/ sad/ angry/ helpless)**
**And positive (e.g. happy) such emotions in the NICU?**
*Probe for various emotions mentioned in Question 2*

**5: What do you feel/think the baby goes through? Do you discuss it with anyone?**
*Probe - Pain?*
*- Discuss with immediate family, peers, neighbours, NICU staff.*

**6. How do you cope when you are faced with this situation?**
*Probe: present condition, knowledge about diagnosis*

**7. Do you have any earlier experiences with seeking care for sick babies?**

*Probe - In your family? Among you friends?*

**8. Let us discuss about the expenses related to your baby's condition. Can you tell me how you and/or your family mobilized the money for your child's treatment?** *(assess if guardian is willing to speak. If yes, continue. If not, reassure him/her and ask if they can answer now. Else, come back again)*

| Source | Yes? |
|---|---|
| It was free | |
| Salary/Savings | |
| Working overtime | |
| Mortgaging of assets (jewellery, land, livestock, etc) | |
| Selling of assets (jewellery, land, livestock, etc) | |
| Borrowed from relatives/friends without interest | |
| Borrowed from relatives/friends with interest | |
| Loan (moneylender, bank) | |
| Health insurance<br>a) BPL free/poor free b) Government employee c) RSBY d) Private Insurance e) NRHM scheme f) ESI g) Any other, specify……… g) None | |
| Others, specify | |

**9. Has the illness affected the family financially? What changes or adjustments did you have to make in your daily life due to your child's illness?**
*Probe- cutting down costs), Work, Household chores, Accommodation, Baby sitting*
*Others- child education, food security, changes in spouse's life*

**Closing questions:**
**10. What is the one thing that is on your mind right now?**

**11. What help could you have received to make this entire experience better?**
*Probe- Health, finances, support (peer, family, health system)*

Is there anything else you would like to tell me? Do you have any questions for me?

-Thank you for your patience and time.

- End of interview-
