# Supplementary material for: Stressors and support system among parents of neonates hospitalised with systemic infections: qualitative study in South India
Source: Arch Dis Child. 2020 Nov 11;106(1):20–9. doi: 10.1136/archdischild-2020-319226 (PMC7788219; doi:10.1136/archdischild-2020-319226)
Supplement: Supplementary data [file archdischild-2020-319226supp003.pdf]

Supplement 3

a. **Reasons for exclusion**

Number of participants	Reason for denial of consent/exclusion
6	Too stressed to talk
2	Repeatedly (more than three times) unavailable for interview despite providing consent
1	Shifted (against medical advice) the baby to a government facility after agreeing to participate
