# Supplementary material for: Stressors and support system among parents of neonates hospitalised with systemic infections: qualitative study in South India
Source: Arch Dis Child. 2020 Nov 11;106(1):20–9. doi: 10.1136/archdischild-2020-319226 (PMC7788219; doi:10.1136/archdischild-2020-319226)
Supplement: Supplementary data [file archdischild-2020-319226supp004.pdf]

Supplement 4: Financing of NICU-related costs

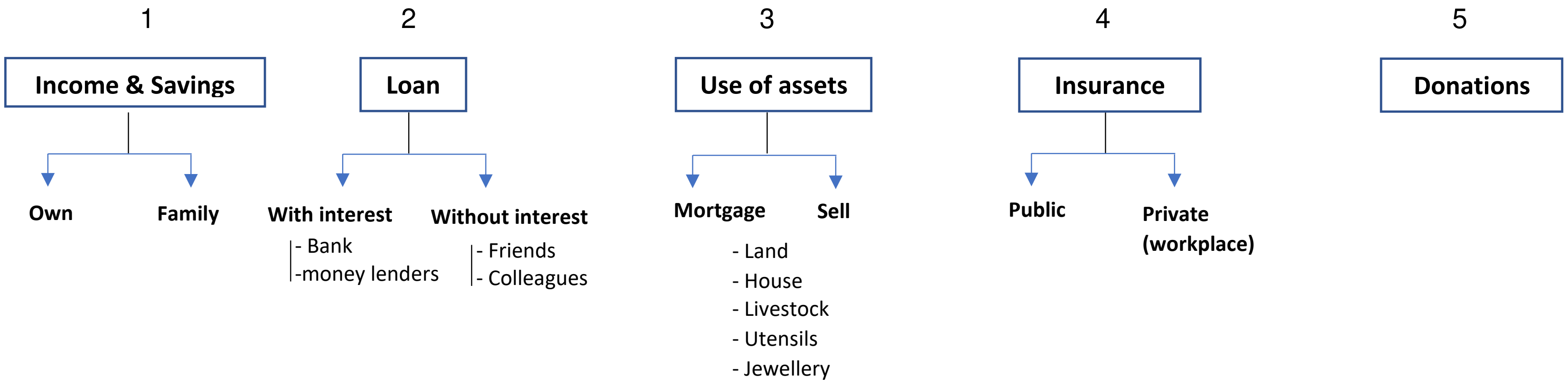

Strategies of financing: There were 3 ways in which families financed for the NICU-related costs:

1. Completely out of pocket: Families paid entirely through out-of-pocket financed through savings and/or income, contributions by relatives and friends, sale or mortgaging of physical assets
2. Combination: Families arranged to pay by a combination of out of pocket payments, acquiring loans, partial insurance cover (private/public) and/or donations from charities or financial trusts (only for those below poverty line).
3. Completely covered by insurance: A final type was where the entire expenses was covered by insurance.
